# Novel CCL21-Vault Nanocapsule Intratumoral Delivery Inhibits Lung Cancer Growth

**DOI:** 10.1371/journal.pone.0018758

**Published:** 2011-05-03

**Authors:** Upendra K. Kar, Minu K. Srivastava, Åsa Andersson, Felicita Baratelli, Min Huang, Valerie A. Kickhoefer, Steven M. Dubinett, Leonard H. Rome, Sherven Sharma

**Affiliations:** 1 Department of Biological Chemistry, David Geffen School of Medicine at University of California Los Angeles, Los Angeles, California, United States of America; 2 University of California Los Angeles Lung Cancer Research Program of the Jonsson Comprehensive Cancer Center, David Geffen School of Medicine at University of California Los Angeles, Los Angeles, California, United States of America; 3 Division of Pulmonary and Critical Care Medicine, Department of Medicine, David Geffen School of Medicine at University of California Los Angeles, Los Angeles, California, United States of America; 4 Molecular Medicine Laboratory, Veteran's Affairs Greater Los Angeles Healthcare System, Los Angeles, California, United States of America; 5 California NanoSystems Institute at University of California Los Angeles, Los Angeles, California, United States of America; University of Windsor, Canada

## Abstract

**Background:**

Based on our preclinical findings, we are assessing the efficacy of intratumoral injection of dendritic cells (DC) transduced with an adenoviral vector expressing the secondary lymphoid chemokine (CCL21) gene (Ad-CCL21-DC) in a phase I trial in advanced non-small cell lung cancer (NSCLC). While this approach shows immune enhancement, the preparation of autologous DC for CCL21 genetic modification is cumbersome, expensive and time consuming. We are evaluating a non-DC based approach which utilizes vault nanoparticles for intratumoral CCL21 delivery to mediate antitumor activity in lung cancer.

**Principal Findings:**

Here we describe that vault nanocapsule platform for CCL21 delivery elicits antitumor activity with inhibition of lung cancer growth. Vault nanocapsule packaged CCL21 (CCL21-vaults) demonstrated functional activity in chemotactic and antigen presenting activity assays. Recombinant vaults impacted chemotactic migration of T cells and this effect was predominantly CCL21 dependent as CCL21 neutralization abrogated the CCL21 mediated enhancement in chemotaxis. Intratumoral administration of CCL21-vaults in mice bearing lung cancer enhanced leukocytic infiltrates (CXCR3^+^T, CCR7^+^T, IFNγ^+^T lymphocytes, DEC205^+^ DC), inhibited lung cancer tumor growth and reduced the frequencies of immune suppressive cells [myeloid derived suppressor cells (MDSC), T regulatory cells (Treg), IL-10 T cells]. CCL21-vaults induced systemic antitumor responses by augmenting splenic T cell lytic activity against parental tumor cells.

**Significance:**

This study demonstrates that the vault nanocapsule can efficiently deliver CCL21 to sustain antitumor activity and inhibit lung cancer growth. The vault nanocapsule can serve as an “off the shelf” approach to deliver antitumor cytokines to treat a broad range of malignancies.

## Introduction

Lung cancer is the leading cause of cancer death in the United States and worldwide [1]. To date with the existing forms of therapy, the overall long term survival is only 15%. Thus, new therapeutic strategies are needed. We are evaluating a novel vault nanocapsule as a proof of concept for the delivery of immune potentiating cytokines for an “off the shelf reagent” for immune based therapy for lung cancer.

Vaults are cytoplasmic ubiquitous ribonucleoprotein particles first described in 1986 [2]. Native vaults are 12.9±1 MDa ovoid spheres with overall dimensions of approximately 40 nm in width and 70 nm in length [3], [4], present in nearly all-eukaryotic organisms with between 10^4^ and 10^7^ particles per cell [5]. Despite their cellular abundance, vault function remains elusive although they have been linked to many cellular processes including the innate immune response, multidrug resistance in cancer cells, multifaceted signaling pathways, and intracellular transport [6].

As a naturally occurring nanocapsule, the vault particle may be an ideal structure to engineer as a therapeutic system for compound encapsulation, protection, and delivery. Vaults are highly stable structures *in vitro*, and a number of studies indicate that the particles are non-immunogenic [7]. Vaults can be engineered and expressed using a baculovirus expression system and heterologous proteins can be encapsulated inside of these recombinant particles using a protein-targeting domain termed INT for vault INTeraction. Several heterologous proteins have been fused to the INT domain (e.g. fluorescent and enzymatic proteins) and these fusion proteins when packaged into the recombinant vaults, retain their native characteristics, and thus confer new vault properties [8], [9].

CCL21 has been identified as a lymphoid chemokine that is predominantly and constitutively expressed by high endothelial venules in lymph nodes and Peyer's patches, lymphatic vessels and stromal cells in spleen and appendix [10]. CCL21 binds to the chemokine receptor CCR7 and is a chemoattractant for mature dendritic cells (DC), naive and memory T cells [11], [12]. This chemokine, along with CCL19, is required for normal lymphoid tissue organization that is ultimately essential for effective T cell-DC interactions. Natural killer (NK) and natural killer T (NKT) antitumor effectors also express CCR7 receptor and are chemo attracted by CCL21. The use of chemokines to attract DC, lymphocyte, NK and NKT effectors into tumors can serve as an effective antitumor strategy. Based on this concept, we have previously shown that intratumoral administration of recombinant CCL21 reduces tumor burden in murine lung cancer models [13]. The antitumor activity induced by recombinant CCL21 however required high and frequent dosing because proteins administered intratumorally are not retained locally for prolonged periods. Although these studies delineated the role of CCL21 as an effective antitumor agent, frequent high dose intratumoral administration is clinically limiting with the potential of unnecessary systemic toxicity. Based on the limitations of this approach, we examined the use of DC for intratumoral CCL21 delivery [14], [15]. In preclinical studies we demonstrated that intratumoral administration of CCL21 gene modified DC led to tumor eradication in murine lung cancer models. Following our initial description of the antitumor activities of CCL21, several groups have reported that CCL21 has potent antitumor properties in a variety of model systems [16], [17], [18], [19], [20]. In all models, CCL21 demonstrated potent regression of tumors, which was shown to be dependent on host T cell immunity.

Based on extensive pre-clinical evaluation, we began an NCI funded clinical trial one year ago assessing the intratumoral injection of DC transduced with an adenoviral vector expressing the CCL21 gene (Ad-CCL21-DC) in a phase I trial in advanced non-small cell lung cancer (NSCLC) [21]. While our clinical studies utilizing intratumoral administration of CCL21 gene modified DC shows immune enhancement, the preparation of CCL21 expressing autologous DC is cumbersome, expensive and time consuming. An efficacious off-the-shelf reagent would facilitate the evaluation of this chemokine-based therapy. Towards achieving this goal, we are evaluating a non-DC based approach which utilizes vault nanoparticles for intratumoral CCL21 delivery for the purpose of initiating antitumor immune responses in lung cancer. We anticipate that the use of vault nanoparticles will circumvent autologous DC preparation, minimize batch to batch variability and allow for comparability and standardization. In addition, vault nanoparticles engineered to release CCL21 can be used to treat a broad range of malignancies.

In this study we evaluated the effect of vault nanocapsule delivery of CCL21 on the growth of Lewis Lung (3LL) tumors *in vivo*. Our findings indicate that a single intratumoral administration of CCL21-vault nanocapsules recruits antitumor effectors, induces potent antitumor activity and inhibits tumor growth.

## Materials and Methods

### Recombinant Vaults

The CCL21 cDNA was fused in frame to either INT or mCherry-INT [22]. Murine CCL21 was PCR amplified with the primers: CCL21- forward GCGCGGATCCCCATGGCTCAGATGATG and CCL21- reverse GCGCAGATCTTCCTCTTGAGGGCTGTGTCTG. To form mCCL21-mCherry-INT in pFastBac, purified CCL21 PCR product was digested with BamH1 and Bgl I and ligated to BamH1 phosphatase treated mCherry-INT pFastBac. Human CCL21 was PCR amplified with the primers: CCL-21 F-SpeI CCCCACTAGTC CAGTTCTCAGTCACTGGCTCTG, CCL-21-NheI CCCCGCTAGCTGGCCCTTTAGGGGTCTGTG, INT with NheI CCCCGCTAGCTGCACACAACACTGGCAGGA, INT with XhoI GGGGCTCGAGTTAGCCTTGACTGTAATGGA to form hCCL21-INT. Recombinant (r) baculoviruses were generated as described [7]. Sf9 cells were infected with mCCL21-mCherry-INT, hCCL21-INT or CP-MVP encoding baculoviruses at a multiplicity of infection (MOI) of 0.01 for 65 h. The infected cells were prepared as described [7]. Lysates containing CP-MVP vault were mixed with lysates containing mCCL21-mCherry-INT or hCCL21-INT and were incubated on ice for 30 min to allow the INT fusion proteins to package inside of vaults. Vault were purified as described [8], resuspended in sterile PBS and protein concentration determined by BCA assay (Bio-Rad, CA). Sample integrity was analyzed by negative stain electron microscopy or Coomassie staining of SDS-PAGE followed by Western blot analysis. CCL21-INT protein in the vault was quantified by densitometric analysis of Western blots against purified recombinant INT protein as standard.

### Antibodies

Primary antibodies for western blot analyses were rabbit anti-MVP antibody (1/1000 dilution) [23] or rabbit anti-VPARP antibody (1/500 dilution) [24] and secondary goat anti-rabbit HRP-antibody (1∶2000 dilution) (Amersham). The CCL21 and CXCR3 (220803) mAb were from R&D, (Minneapolis, MN), primary immunostaining CD3 mAb was from DAKO (Cytomation, Carpinteria, CA, USA). FlTC, PE, APC, PerCP or PerCP-Cy7-conjugated anti-mouse mAbs to CD3 (145-2C11), CD4 (RM4-5), CD8α (53-6.7) and isotype control antibody were from BD Biosciences (San Diego, CA), mAbs to detect Tregs with CD4 (GK1.5), CD25 (PC61), intranuclear Foxp3 (FJK-16s) IL-10 (JES5-16E3), IFNγ (XMG1.2), DEC205 (205yekta), CCR7 (4B12) and EpCam (G8.8) were from eBioScience(San Diego, CA), mAb CD11b (M1/70), Gr1 (RB6-8C5), were from BioLegend (San Diego). Bradford protein quantification dye was from Sigma.

### Chemotaxis assay

Chemotaxis assays were performed using 24-well plates with 3 µm pore size inserts (Costar/Corning, Corning, New York) according to the manufacturer's instructions. 2.0×10^5^ T2 cells in serum-free medium were loaded in the upper chamber. 200 ng/ml of CCL21-mcherry-INT/vault, 600 ng/ml rCCL21 (R&D), 200 ng/ml CCL21-mCherry-INT/CP-MVP vault with neutralizing CCL21 antibody (5 µg/ml), 600 ng/ml CCL21 with CCL21 antibody (5 µg/ml) were added to the lower chambers. After 2 hr incubation, migrated cells were enumerated by FACS.

### Antigen processing and presentation assay

DC2.4 (5×10^4^c/well) were plated in 96-well plates with OVA protein (350 µg/ml), CD8 T cell line B3Z (10^5^c/well), in the presence of control vaults (200 ng/ml), or mCCL21-vaults (200 ng/ml) or rCCL21 (200 ng/ml) for 24 hrs. To determine the impact of CCL21 on APC activity, CCL21 was neutralized with anti-CCL21 Ab (5 µg/ml). IL-2 secreted by activated CD8 T cells was quantified by ELISA (eBioScience).

### Cell culture

The Lewis lung carcinoma cell line (3LL) was obtained from ATCC (Manassas, VA) and was cultured as described [25]. The cell line was mycoplasma free and utilized before the tenth passage.

### Tumorigenesis Model

Pathogen-free C57BL/6 mice or UBC-GFP/BL6 (6–8 wk old; Jackson Laboratory) were maintained in the Veterans Affairs Animal Research vivarium in accord with the institution's animal review board guidelines. All animal work was conducted in accord with the Veterans Affairs Institutional Animal care and Use Committee guidelines: id A3002-01. The institutional review board approved all the studies involving animals in this manuscript. Animals exhibiting signs of pain or meeting the endpoint criteria were euthanized immediately according to the accepted institution based protocol. 3LL tumor cells (1.5×10^5^) were injected *s.c*. in the right suprascapular area of mice. Mice bearing 9-day-old established tumors were treated with a single intratumoral injection of mCCL21-mCherry-INT/CP-MVP vaults (200 ng), CP- MVP vaults (200 ng) in 200 µl or NS diluents. Tumor volumes were monitored and calculated as described [25]. To determine the lymphocytes infiltrating the tumors, UBC-GFP/BL6 mice bearing 9-day tumors were treated and 7 days post treatment, non-necrotic tumors were isolated and frozen in OCT. The frozen tissue was sectioned, fixed and counterstained with 4′,6-diamidino-2-phenylindole (DAPI) and observed under a 1×71 Olympus fluorescence microscope. The images were acquired using the Image Pro software.

### Orthotopic model

3LL-GFP cells (5×10^3^) were implanted in the left lung as described [25]. One week following tumor inoculation, mice were treated with diluent, control vaults or mCCL21-vaults *via* transthoracic injection. Four weeks after tumor implantation, lungs were harvested for evaluation of tumor burden and leukocytic infiltrates as described [25].

### Immunostaining

Tumors section were prepared according to standard protocol [26]. Slides were incubated with primary antibody (CD3 1∶200) overnight at 4°C, washed and incubated with secondary biotinylated. The slides were developed with Vectastain ABC-AP kit and Vector Red substrate solution (Vector Laboratories, Burlingame, California). Slides were counterstained with hematoxylin.

### Flow Cytometry

FACs analyses was performed for CD3, CD4, CD8, CCR7, CD11b, Gr1, DEC205, CD25, FOXP3, CXCR3 and intracytoplasmic T cell IFNγ and IL-10 on a single lung tumor cell suspension as described [25].

### T cell Cytolysis

Splenic T lymphocyte lysis was evaluated against autologous 3LL and control B16 melanoma cells as described [25].

### Statistical analysis

Statistical analyses were performed using t-tests or Mann-Whitney tests. All statistical analyses were done at GraphPad PRISM 5 software.

## Results

### Packaging CCL21 into recombinant vaults

**Figure 1:**
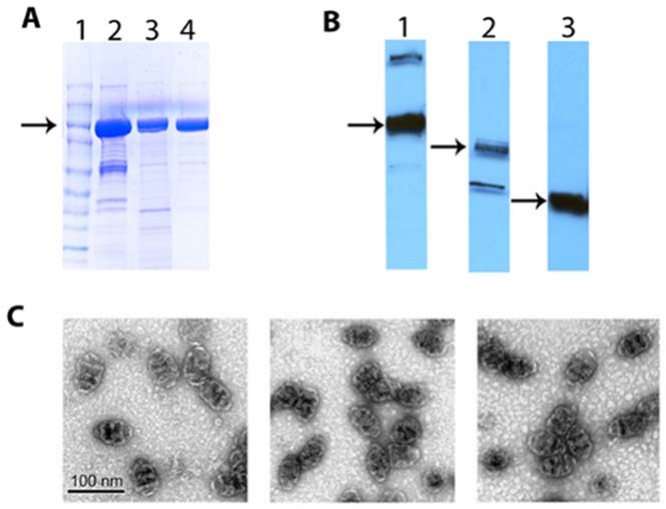
Evaluation of recombinant CCL21-vaults. A. Coomassie stain of purified vaults fractionated on a 4–16% SDS-PAGE. Lane 1, molecular weight markers. Lane 2, mCCL21-mCherry-INT/CP-MVP vaults. The 97-kDa MVP band is indicated by the arrow. Lane 3, hCCL21-INT/CP-MVP vaults. Lane 4, CP-MVP vaults. B. Western blot analyses of purified vaults to confirm the presences of MVP or the indicated INT fusion proteins (arrows). Lane 1, mCCL21-mCherry-INT/CP-MVP vaults were immunoblotted with anti-MVP antibody. Lane 2, mCCL21-mCherry-INT/CP-MVP vaults were immunoblotted with anti-INT antibody. Lane 3, hCCL21-INT/CP-MVP vaults were immunoblotted with anti-INT antibody. C. Negative-stained electron micrographs of purified vaults. CP-MVP vaults (left), mCCL21-mCherry-INT/CP-MVP vaults (center), hCCL21-INT/CP-MVP vaults (right).

Several exogenous proteins have been packaged inside of recombinant vaults (luciferase, green fluorescent protein, mCherry fluorescent protein, adenovirus protein VI, and the major outer membrane protein of Chlamydia) when fused to a 162 amino acids vault-targeting domain (INT) derived from the vault-associated protein (VPARP, amino acids 1563–1724). These fusion proteins bind with high affinity to the interior of the vault particle, are protected from the external environment, and retain their native properties, thus conferring new characteristics onto the recombinant vault particles [7], [9], [22]. The mouse chemokine CCL21 was fused to INT to create a CCL21 fusion protein (mCCL21-INT) that can be packaged inside of recombinant vaults. Mixing of lysates containing recombinant CCL21-INT and rat MVP generated in Sf-9 cells allowed the formation of a macromolecular vault complex containing the CCL21 fusion protein that could be isolated by density gradient ultracentrifugation. The purified vaults contained both MVP as well as CCL21-INT (henceforth referred to as CCL21-vaults) as indicated by Coomassie staining and immunoblot analyses (Fig. 1). For these studies three different vaults were prepared. One was packaged with mouse CCL21 fused to the fluorescent protein mCherry-INT (mCCL21-mCherry-INT/CP-MVP vaults, abbreviated here mCCL21-vaults), a second vault was packaged with human CCL21-INT (hCCL21-INT/CP-MVP vaults, abbreviated here hCCL21-vaults) and the third was a control empty vault (CP-MVP vaults [27]) (Fig. 1A and B). We estimated the amount of CCL21 packaged inside the vaults by quantitative Western blots. Our analysis indicated that 20–30 molecules of the CCL21-INT protein were packaged in each CCL21-vault particle. This is consistent with our previous experience in packaging multiple copies of other INT fusion proteins into recombinant vaults [22]. With an estimate of 20–30 CCL21-INT proteins per vault, it is likely that this is at or near a saturating level for the packaging of this size protein. The CCL21-vaults also exhibited a very similar sedimentation profile on sucrose gradients as vault particles containing the INT domain fused to luciferase, [7], [9], [22] suggesting that incorporation of CCL21-INT did not impact the normal structure of recombinant vault particles. To verify this, we examined purified vault complexes by negative stain transmission electron microscopy (Fig. 1C). The CCL21-vaults exhibited the characteristic barrel shaped morphology of vaults, consistent with the previously established morphology of vaults containing recombinant-INT fusion proteins [9], [27].

### CCL21-vaults are biologically active and induce the migration of T2 cells and enhance DC APC activity

**Figure 2:**
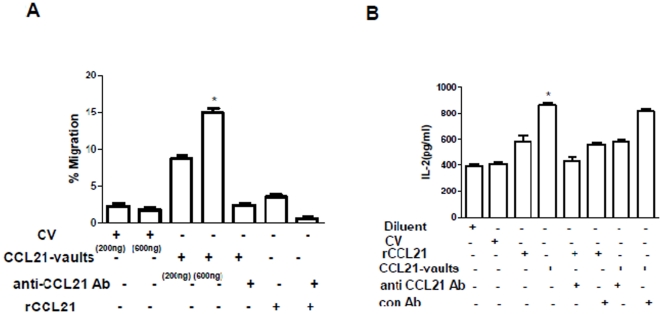
CCL21-vaults enhance the chemotactic migration of T cells and DC APC activity. A. CCL21-vaults increased the migration of T2 cells. 2.0×10^5^ T2 cells were plated in serum-free medium in the upper chamber. mCCL21-mCherry-INT/CP-MVP vaults (200 ng/ml or 600 ng/ml), recombinant CCL21 (600 ng/ml), control vaults (CP-MVP vaults 600 ng/ml) or neutralizing anti-CCL21 recombinant antibody (5 µg/ml) were added to the lower chamber of the wells. Following 2 hr incubation, migration of T2 cells were analyzed by flow cytometry. mCCL21-vaults effectively increased the T2 migration as compared with control. Anti-CCL21 neutralizing Ab to CCL21 abrogated the increase in T2 migration suggesting that CCL21 in the vault predominantly mediate the chemotatic migration of T2 cells. Data in the panel are representative of 2 independent experiments. Bars; Mean ± SEM, *p<0.05 between the mCCL21-vaults and control vaults or anti-CCL21 antibody treatment groups. B. mCCL21-vaults enhanced DC APC activity and blocking CCL21 reversed the increase in APC activity. B3Z cells (1×10^5^ cells/200 µl/well) were co-cultured with DC 2.4 (5×10^4^ cells/200 µl/well) and ovalbumin (350 µg/ml) in the presence or absence of mCCL21-vaults (200 ng/ml) and anti-CCL21 antibody (5 µg/ml) or control antibody (5 µg/ml goat IgG) for 24 hrs. Control vaults were used at a concentration of 200 ng/ml. T cell activation status was measured by IL-2 production by ELISA. Data are representative of 2 independent experiments. Bars; Mean ± SEM, *p<0.05 between the mCCL21-vaults and control vaults or anti-CCL21 antibody treatment groups.

A chemotaxis assay was used to determine if CCL21 retains biological function when packaged in the recombinant vaults. The chemotactic activity of CCL21 is mediated through its receptor CCR7 to induce the migration of T cells and DC. To evaluate the biological activity of CCL21 in the vault, we used T2 hybridoma cells that constitutively express CCR7. Two different concentrations of CCL21-vaults (200 ng and 600 ng), empty vaults (600 ng), and recombinant CCL21 (600 ng) were placed in the bottom chamber of a 24-well transwell plates and 2×10^5^ T2 cells were added to the upper chamber. The number of cells that migrated to the lower chamber was determined by flow cytometry and represented as % migration (Fig. 2A). More than 7.5% of the T2 cells responded to 200 ng of mCCL21-vaults compared with ≤2.5% of the T2 cells incubated with 600 ng of recombinant CCL21. This is a potent response considering that the concentration noted above is for CCL21-vaults and the actual concentration of CCL21 in the vaults is estimated to be ≤20 ng. The increased bioactivity of mCCL21-vaults may be due to increased stabilization of CCL21 resulting from packaging of the protein into the protective environment of the vault lumen. As the fusion protein non-covalently associates within vaults, the vault breathing in solution may release CCL21 in a gradient fashion. Thus the number of cells migrating in response to mCCL21-vaults was higher than the recombinant CCL21 because a steeper gradient is formed. To determine if the migration of T2 cells was CCL21-dependent, an anti-CCL21 neutralizing antibody was utilized to block the chemotactic activity of both recombinant CCL21 and mCCL21-vaults. The anti-CCL21 blocked mCCL21-vault functional activity indicating chemotactic activity in a CCL21 specific manner (Fig. 2A). The impact of mCCL21-vaults on DC APC activity was evaluated *in vitro*. In comparison to control vaults, mCCL21-vaults augmented DC capacity to process and present ovalbumin and activate CD8 T cells to secrete IL-2. Neutralization of CCL21 abrogated the increase in DC APC activity to control levels (Fig. 2B). Similar results were observed with hCCL21-vaults on DC APC activity (data not shown).

### CCL21-vaults enhance the recruitment of leukocytic infiltrates and inhibit 3LL tumor growth

**Figure 3:**
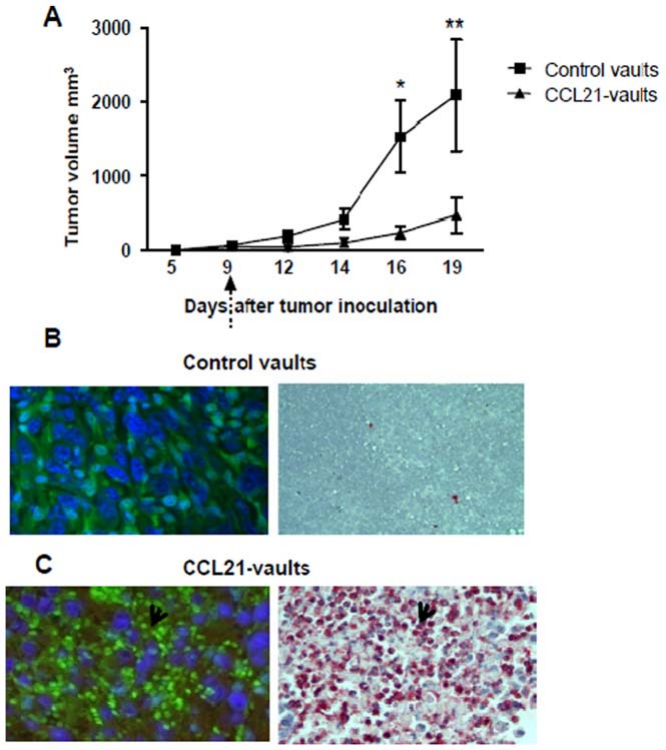
Intratumoral CCL21-vaults reduce tumor growth and enhance T cell leukocytic infiltrates. A. CCL21-vaults inhibited tumor growth and enhanced intratumoral T cell infiltrates in lung cancer. C57BL/6 mice bearing 9 day 3LL established tumors (*s.c.*) were treated intratumorally with control vaults (200 ng), mCCL21-vaults (200 ng) or diluent normal saline (NS). Bisecting tumor diameters were measured with calipers. B–C. mCCL21-vaults increased the influx of CD3 expressing T cells in the tumor as compared to control vaults. UBC-GFP/BL6 mice bearing 9-day established tumors were treated intratumorally with control vaults or mCCL21-vaults. OCT frozen tissue was sectioned, fixated onto slides, and counterstained with the nuclear dye DAPI (blue). Control tumors (*top panel*) demonstrate very limited green fluorescence (infiltrating cells) under a high-power view (magnification, ×400). Most green fluorescent cells are large polygonal stromal cells while tumor cells are counterstained blue by DAPI. Immune staining for T cells shows very few CD3 expressing T lymphocytes (top right panel). In contrast, prominent tumor-infiltrating lymphocytes, small round green fluorescent cells are evident in tumors from the mCCL21-vault-treated mice (arrow, *bottom panel*). Immune staining for T cells shows that the infiltrates are CD3 expressing T cells (bottom right). Data; Mean ± SEM, *p<0.05 between mCCL21-vault and control group, n = 10 mice/group.

**Figure 4:**
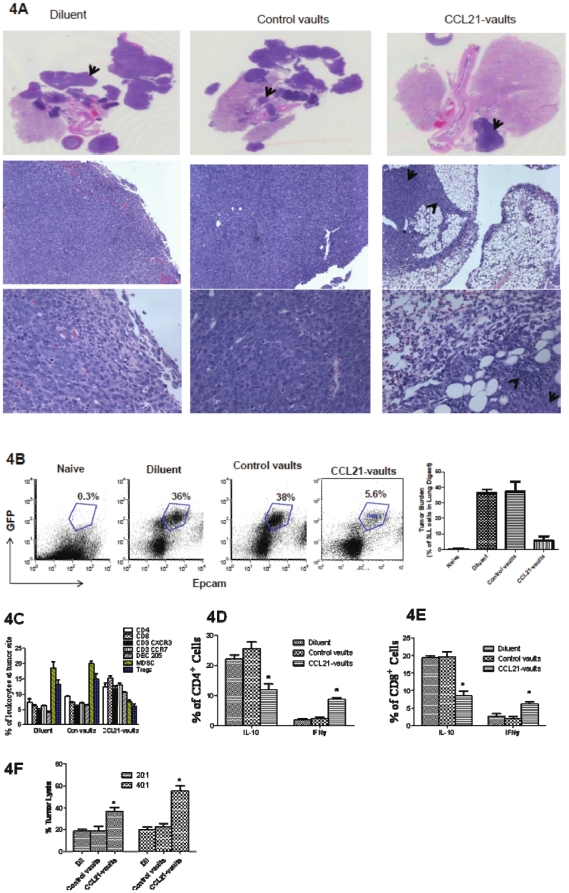
CCL21-vaults reduced tumor growth and immune suppressors (MDSC and Tregs) and enhanced intratumoral immune cell infiltrates in 3LL orthotopic lung cancer model. 5×10^3^ 3LL-GFP tumor cells were injected in the left lung via transthoracic route. One week after tumor injections, mice were treated with diluent (NS), control vaults (2 µg) or mCCL21-vaults (2 µg) via transthoracic route in the left lung. Day 28 post tumor implantation, lungs were harvested for the analysis of tumor burden and leukocytic infiltrates. A. H&E staining of lung tumor sections from diluent or control vaults showed increased tumor masses (tumor mass depicted by arrow top panel and infiltrates bottom right panel) as compared to reduced tumor mass in the CCL21-vault treatment group. B. Tumor burden was calculated on total percentage of GFP and Epcam expressing tumor cells in total lung digest. Naïve lung was used as control to set up the gate. CCL21-vault treatment reduced tumor growth. C. CCL21-vaults augmented CD4, CD8, CXCR3^+^CD3^+^T, CCR7^+^CD3^+^T and DEC205^+^DC infiltrates and had reduced frequencies of MDSC and Tregs. D–E. The CCL21-vault treated mice had increased tumor infiltrates with high IFNγ but reduced IL-10 expressing T cells compared to controls. F. CCL21-vault treatment enhanced the cytolytic activity of purified splenic T cells against parental 3LL tumors *in vitro* (E∶T of 20∶1 and 40∶1). Data bars, mean ± SEM, *p<0.05 between CCL21-vaults and control vault groups, n = 10 mice/group.

We determined the antitumor activity of mCCL21-vaults on established 3LL tumor *in vivo*. A single intratumoral injection of mCCL21-vaults (200 ng) led to significant decrease in tumor growth compared to empty vaults (Fig. 3A). The mCCL21-vault treatment group showed enhanced intratumoral leukocytic infiltrates compared to control vaults (Fig. 3B) and immune staining demonstrated that the infiltrates were predominantly CD3 expressing T cells (Fig. 3C bottom right panel). The antitumor efficacy of mCCL21-vaults was determined in the 7-day established orthotopic 3LL lung cancer model. mCCL21-vaults inhibited tumor burden by 7-fold compared to controls (Fig. 4A–B). In the control treatment groups (diluents or control vault), there were 7–9% decrease in the average body weight at the end of the experimental duration but no significant weight change was observed in the CCL21-vault treatment group. An evaluation of intratumoral leukocytic populations showed enhanced frequency of CD4, CD8, CD3 CXCR3, CD3 CCR7 and DEC205 but reduced frequency of MDSC and Tregs (Fig. 4C).

### CCL21-vaults increase tumor infiltrating T cells with enhanced IFNγ but reduced IL-10 and augment systemic T cell cytolytic activity

Tumor T lymphocytic infiltrates from mCCL21-vault treated mice had increased intracytoplasmic IFNγ and reduced IL-10 (Fig. 4D–E). Intratumoral mCCL21-vaults induced systemic antitumor immune activity: splenic T cells from mCCL21-vault treated mice had augmented lytic activity against parental tumor cells *in vitro* (Fig. 4F).

## Discussion

We have previously shown that intratumoral recombinant CCL21 or CCL21 gene modified DC induce a T cell dependent tumor reduction in murine models of lung cancer [13], [14], [15],[28],[29]. Based on extensive preclinical modeling, we have translated our intratumoral DC-AdCCL21 approach to treat advanced stage lung cancer in a phase I clinical trial. Although the initial results of this trial suggest immune enhancement, we are evaluating CCL21 delivery mechanisms that will obviate the need to isolate and culture autologous DC. To this end we evaluated CCL21-vault nanocapsules as an “off the shelf” therapeutic platform against tumor growth *in vivo* utilizing the well characterized 3LL cancer model. Our rationale for administration of intratumoral CCL21-vault nanocapsules is to promote the recruitment of T lymphocytes and DC into the tumor microenvironment for a robust antitumor activity.

As a naturally-occurring, non-immunogenic, protein-based nanoscale capsule, the vault particle has the potential to serve as a flexible therapeutic delivery vehicle. Engineered in the baculovirus system, the particle self-assembles from a single expressed protein which can be modified to allow cell targeting, specific endocytosis and endosome penetration [7], [8], [9], [30]. Proteins and peptides fused to a vault targeting domain are packaged in the particle, protected from the external environment and released slowly. This is the first study to describe that vault nanocapsules can be engineered to package and retain biologically active CCL21. Our results demonstrate that although empty vaults and rCCL21 are each chemotactic, packaging the chemokine into vault synergistically enhanced T cell migration, suggesting that the sustained release of CCL21 by the vault particle can establish a steep chemotatic gradient. Neutralizing antibodies to CCL21 abrogated the chemotactic activity of the CCL21-vault demonstrating that the chemotactic activity was predominantly CCL21 dependent. Based on the results of chemotactic experiments, we evaluated the impact of CCL21-vaults on DC APC activity. CCL21-vaults augmented DC APC activity and neutralization of CCL21 abrogated this activity. In comparison to rCCL21, CCL21-vaults stimulated DC APC activity to greater levels at one tenth the concentration of rCCL21, suggesting a greater DC affinity for the CCL21-vault nanocapsule.

Based on our *in vitro* findings, CCL21-vaults were evaluated in a therapeutic lung cancer model *in vivo*. Our results demonstrate that the vault design is effective at inhibiting the growth of established tumors. A single intratumoral injection of CCL21-vaults led to significant inhibition in tumor growth compared to controls. Our data suggests that the CCL21-vault formulation is as effective in comparison to the frequent high doses of rCCL21 [13]. In both the subcutaneous and orthotopic lung tumor models the CCL21-vault therapy was administered intratumorally. In future studies when evaluating the efficacy of tumor antigen specific vault nanocaspsules vaccination routes in the protective and therapeutic settings that include *i.v* or *i.p.* delivery routes, changes in plasma/serum transaminase level will be quantified for vault effects on liver function. Although in the migration assay, the control vaults demonstrated chemotactic activity, control vaults did not affect tumor growth compared to the diluents treatment group. This suggests that CCL21 is the critical component in the vault nanocapsule for the induction of antitumor activity. In future work we will screen both the pro-apoptotic and anti-apoptotic proteins in the tumors to determine changes in response to CCL21-vault nanocapsule therapy to mechanistically explore the apoptotic programs induced by this therapy. *In vivo*, human CCL21 containing vaults were as effective as the murine CCL21-vault in reducing 3LL tumor growth (data not shown). In comparison to controls, CCL21-vault treatment resulted in extensive tumor T and DC leukocytic infiltrates. Intratumoral infiltration by elevated frequencies of T lymphocytes [31], [32] and APC [33] in lung cancer is associated with a better patient outcome. There was an increased IFNγ and reduced IL-10 cytokine signature of T lymphocytes infiltrating the tumors of CCL21-vault treated mice. Based on our previous observations with rCCL21 in preclinical models [13], the increased frequency of T cell subsets (CD4 and CD8), CXCR3 and CCR7 expressing T cell effectors and DC in the lung tumor microenvironment is beneficial and most probably responsible for enhanced antitumor activity of CCL21-vaults. In a recent study, Dieu-Nosjean et al. [33] retrospectively identified ectopic lymph node or tertiary lymphoid structures within human non-small cell lung cancer specimens and demonstrated a correlation of the cellular content with clinical outcomes. The density of DC-Lamp+, mature DC within these structures was a predictor of long-term survival in the selected lung cancer patient population. The authors observation that a low density of tumor-infiltrating CD4^+^ and T-bet^+^T lymphocytes were present in tumors poorly infiltrated by DCLamp^+^ mature DC provides additional supportive evidence for the prognostic importance of an adaptive immune reaction to a solid tumor [33]. In the present study we do not know if the involvement of CD1d-mediated antigen presentation to NKT cells contributes to the antitumor activity of CCL21-vault nanocapsules. However since NKT cells express the CCR7 receptor, future work will delineate their role in CCL21-vault nanocapsule mediated antitumor reactivity.

Immune suppression has a crucial role in promoting tumor progression. Treg and MDSC contribute to immune suppression in the tumor bearing host. Although T cells accumulate in lung cancer tissues they fail to respond because a high proportion of tumor infiltrating lymphocytes (TIL) are Tregs [34], [35]. Treg cells actively down regulate the activation and expansion of antitumor reactive T cells [36], [37], [38] and NK cells [39]. In addition to Tregs, MDSC have a strong suppressive activity in cancer patients [40], [41], [42], [43] and tumor-bearing mice [44], [45], [46]. These immune suppressive cells inhibit T cell [47], [48] and NK cell activity [49], [50] and pose a significant hurdle to successful immune based therapy. The reduction in these immune suppressor phenotypes may enhance immune based anti tumor approaches. We found that in comparison to controls, CCL21-vault therapy group had reduced the frequencies of Treg and MDSC and systemically enhanced T lymphocytic lysis of parental tumor cells. Thus the benefit of utilizing CCL21-vaults is the recruitment and activation of antitumor effectors and reduced frequencies of Treg and MDSC immune suppressors.

Our combined results indicate that the CCL21-vault nanocapsule platform is an effective antitumor strategy. This holds significance for broad application as an off the shelf reagent for cancer therapy. We have demonstrated that vault nanoparticles containing the major outer membrane protein of Chlamydia, could be delivered to the respiratory tract, by the intranasal route and induced robust anti-chlamydial immune responses [7]. The addition of EGF onto the outside of vault particles can be used to specifically target cells expressing high levels of EGFR and leads to phosphorylation of Tyr1173 on the receptor [30]. This suggests that vault particles can be designed as multifunctional vehicles that can be further engineered for target specific delivery as well as carriers of specific payloads that can act as tumor antigens to prime the immune system to potentially act as vaccines to prevent tumor recurrence and metastasis. These multifunctional nanoparticles may prove indispensable as cancer therapeutics. The results of our study are encouraging and warrant further evaluation of the vault nanocapsule delivery platform for its full therapeutic potential in lung cancer and other malignancies.
